# Diagnostic distribution of 100 strictly unilateral headaches consulting in a specialised clinic

**DOI:** 10.1186/1129-2377-14-S1-P41

**Published:** 2013-02-21

**Authors:** C Ramon, G Mauri, J Vega, M Para, M Rico, G Moris, J Pascual

**Affiliations:** 1 University Hospital Central de Asturias, Spain

## Introduction and objectives

Pain location is an important point in the diagnosis of headaches. Our aim was to analyse the diagnostic distribution of the first 100 patients consulting in our specialised headache clinic due to strictly unilateral headache.

## Patients and methods

Headache diagnoses for all patients sent to our headache clinic in the last year and referring to strictly unilateral headaches were analysed according the current ICH-II classification. We receive patients aged 14 or older.

## Results

The 100 collected patients with strictly unilateral headaches accounted for the 18.9% of the 528 patients seen in our clinic in the study period. Strictly unilateral headaches were more frequent in males (58%). Age ranged from 19 to 81 years. Diagnostic distribution was as follows: cluster headache (38 cases)> a variety of secondary headaches (14 cases)> migraine (11 cases)> cervicogenic headache (9 cases)> hemicrania continua (8 cases)> nummular headache (6 cases)> psychogenic headache (5 cases)> paroxysmal hemicrania (4 cases)> SUNCT syndrome (3 cases)> stabbing headache (1 case)> and probable hypnic headache (1 case).Mean, median and mode of age at onset felt between 47 and 58 years for several diagnoses (cervicogenic headache, nummular headache, psychogenic headaches, hemicrania continua and paroxysmal hemicrania), between 25 and 35 years for cluster headache, below 25 for migraine and, in general, were older than 55 for secondary headaches.

## Conclusions

Strictly unilateral headaches account for almost 20% of headaches attending a headache clinic. Trigemino-autonomic headaches in general (52%) and cluster headache in particular (38%) are the most frequent diagnosis, but, if we include cervicogenic headaches, secondary headaches are diagnosed in one out of five cases. Age can be of important help in their presumptive diagnosis. Supported by the PI11/00889 FISSS grant (ISCIII).

